# The potential of in ovo-fed amino acids to alleviate the effects of heat stress on broiler chickens: effect on performance, body temperature, and oxidative status during the finisher phase

**DOI:** 10.1016/j.psj.2024.103821

**Published:** 2024-05-09

**Authors:** Moustafa Yehia, Angel Rene Alfonso-Avila, Jean-Michel Allard Prus, Véronique Ouellet, Nabeel Alnahhas

**Affiliations:** ⁎ Department of Animal Science, Faculty of Agricultural and Food Sciences, Université Laval, Quebec City G1V 0A6, Quebec, Canada; † Deschambault Research Center in Animal Science (CRSAD), Deschambault G0A 1S0, Quebec, Canada; ‡ Swine and Poultry Infectious Diseases Research Center, Université de Montréal, Saint-Hyacinthe J2S 2M2, Quebec, Canada; § Scott Hatchery, Scott G0S 3G0, Quebec, Canada

**Keywords:** broiler, heat stress, in ovo feeding, amino acid, thermoregulation

## Abstract

The aim of the current study was to investigate the potential of in ovo-fed amino acids (**AA**) to reduce the effects of heat stress on finishing broiler chickens. To achieve this, a total of 1,400 fertile hatching eggs were randomly distributed into 5 groups (n = 280/group) and injected with one of the following in ovo treatments on embryonic day 18: 52 µL of sterile diluent/egg (**CTRL**), CTRL + 1.0 mg of L-Leucine (**T1**), CTRL + 0.45 mg of leucine + 1.15 mg of methionine (**T2**), CTRL + 3.0 mg of methionine + 2.0 mg of cysteine (**T3**), and CTRL + 0.40 mg of leucine + 1.60 mg of methionine + 1.60 mg of cysteine (**T4**). After hatch, chicks were allocated according to a complete randomized block design comprising 2 thermal conditions: thermoneutral (24°C, 45% RH) and heat stress (34°C, 55–60% RH) with 5 pens/group/condition. The cyclical heat stress regimen (10 h/d) was then applied from d 29 to d 34. Compared to the CTRL group, T3 and T4 exhibited a higher BW during the starter phase (*P* < 0.001). T4 also had a lower feed conversion ratio (**FCR**) than CTRL during this same phase (*P* = 0.03). During the grower phase, males of all treatment groups consistently exhibited higher BW compared to the CTRL group, which was not observed among female birds (*P_Sex × TRT_* = 0.005). During the finisher phase, the in ovo treatment effect on performance was not significant. However, heat-stressed birds from treatment group T3 and T4 exhibited lower facial temperatures (*P_day × TRT_* < 0.001) as well as lower plasma (*P_condition x TRT_* = 0.039) and liver (*P_condition x TRT_* < 0.001) malonaldehyde concentrations compared to the CTRL group. In conclusion, in ovo-fed AA have the potential to modulate the effects of heat stress on finishing broiler chickens by limiting its detrimental consequences, including increased body temperature and oxidative damage.

## INTRODUCTION

Broiler meat continues to increase in popularity. As of 2020, it represented 41% of global meat consumption and it is expected to account for 52% of all consumed meat worldwide by 2030 (OECD, 2022). In developed countries, this increased popularity of broiler meat is driven by its perception as a healthy meat (i.e., high in protein and low in fat). While in developing countries, this popularity is mainly driven by its comparatively lower price compared to meat from other animal species (OECD, 2022). To meet the increasing global demand for broiler meat, the poultry industry utilizes genetic selection to create and develop broiler strains of faster growth rates, higher feed efficiency, and greater breast meat yield (Zuidhof et al., 2014). Improved growth rate is associated with increased metabolic rate and increased metabolic heat production (Nascimento et al., 2017). These selection-induced physiological changes, coupled with the inherent biological characteristics of chickens, such as the absence of sweat glands in their skin and the insulating capacity of their feathers, have increased broilers’ susceptibility to heat stress (Tabler et al., 2020; Malila et al., 2021). In addition to these distinctives physiological characteristics in chickens, the escalating surface temperatures due to global warming have introduced heat stress as a prominent challenge for the poultry industry. As a consequence, anticipated economic repercussions for the poultry sector are expected to worsen in the coming years (Kumar et al., 2021).

Several recent reviews comprehensively summarized the impact of heat stress on poultry production (Lara and Rostagno, 2013; Wasti et al., 2020; Liu et al., 2020a; Nawaz et al., 2021). Exposure to ambient temperatures above the upper critical limit of the thermoneutral zone has been shown to impair growth performance in modern broiler strains by reducing weight gain due to decreased feed intake (Goo et al., 2019; Awad et al., 2020). Heat stress also deteriorates the quality of broiler breast meat by accelerating post-mortem acidification and inducing oxidative damage in the pectoral muscles (Zaboli et al., 2019). It also diminishes the capacity of the immune system to produce an effective immune response (Hirakawa et al., 2020; Monson et al., 2018). Additionally, heat stress induces changes in gut microbiota by promoting the proliferation of potentially opportunistic pathogenic bacteria such as *Clostridium* and reducing beneficial bacteria such as *Lactobacillus* and *Bifidobacterium* (Rostagno, 2020). Diminished immune function, elevated levels of harmful bacteria, and increased intestinal barrier permeability (Nanto-Hara et al., 2020; Tabler et al., 2020) render broilers more susceptible to diseases, amplifying economic losses due to high mortality rates.

Multiple strategies have been proposed to alleviate the adverse impact of heat stress on broiler production. These approaches include the management of environmental conditions in poultry houses using mechanical cooling systems and sprinklers (Liang et al., 2020). Additionally, alternative feeding strategies both quantitative (e.g., feed restriction, dual feeding, and wet feeding) and qualitative (e.g., high energy diets and supplementation with amino acids) have been suggested (Teyssier et al., 2022). Moreover, thermal conditioning during embryogenesis or early post-hatch has been explored as another potential strategy (Madkour et al., 2022). In recent years, there has been a growing interest in the use of in ovo feeding with different nutrients to mitigate the negative effects of heat stress. In ovo feeding is a technique that involves injecting nutrients into fertilized eggs during embryogenesis to enhance physiological performance. In a series of studies, the potential of branched-chain amino acids (**BCAA**) to improve thermal tolerance in chickens has been investigated (Han et al., 2017; Han et al., 2018; Han et al., 2019; Han et al., 2022; Han et al., 2020; Han et al., 2021). These researchers injected sterile water (500 µL/egg), L-Leucine (L-Leu, 35 mmol/egg), L-Isoleucine (L-Ile, 21 mmol/egg) and L-Valine (L-Val, 29 mmol/egg) in the yolk sac of fertile eggs of a Japanese Chunky broiler breed on embryonic day (**ED**) 7. On d 5 posthatch, male chicks were exposed to heat stress (35°C for 3 h) and their rectal temperatures (**RT**) were recorded at 1 h intervals. Chickens from the L-Leu-injected group had significantly lower RT compared to the control group (Han et al., 2017). The role of L-Leu in thermoregulation and its hypothermic effect in chickens were both confirmed in a later study by the same researchers (Han et al., 2019).

In addition to the detrimental impacts of elevated core body temperature, oxidative stress in multiple body organs is one of the most harmful consequences of exposure to high ambient temperatures (Azad et al., 2013; Del Vesco et al., 2017). Sulfur-containing amino acids (**SAA**; Methionine and Cysteine) are known for their antioxidant properties through their modulatory activities on the glutathione-related antioxidant enzymes (Tesseraud et al., 2008). The potential of in ovo-fed SAA to alleviate oxidative damage induced by heat stress during embryogenesis has also been investigated (Elwan et al., 2019). In this study, fertile Ross broiler breeder eggs were exposed to 39.6°C for 6 h/d from ED 10 to ED 18 and injected on ED 17.5 with a combination of L-Methionine and L-Cysteine (5.90 mg L-Met and 3.40 mg of L-Cys) or with saline solution (injected control) through the air chamber. The newly hatched chicks from SAA-injected eggs exhibited significantly elevated levels of glutathione and greater total antioxidant capacity in samples from the heart, liver, kidneys, serum, and small intestine. In a similar but more recent study, fertile eggs from a commercial strain were injected with 3.5 mg of L-Cys at ED 17.5 after exposure to 39.6°C for 6 h/d from ED 10 to ED 17.5 (Ajayi et al., 2022). Compared to the control group, day-old chicks that hatched from the L-Cys-injected eggs had significantly lower concentrations of malondialdehyde (**MDA**) and higher superoxide dismutase activity. This indicates a more efficient response of the antioxidant defense system in chicks from treated eggs. The data derived from the aforementioned studies suggest that in ovo feeding of BCAA and SAA represents a promising approach that could be used in complement with other strategies to further alleviate the detrimental effects of heat stress on poultry production. However, most of these studies have only examined the use of these amino acids under laboratory conditions or were limited to the early posthatch phase. Hence, the effects of in ovo feeding of these amino acids during the finisher phase, where birds are the most susceptible to heat stress due to higher BW (Liu et al., 2020a; Andretta et al., 2021), have yet to be investigated. Moreover, in these studies, amino acids were manually injected into the eggs, which considerably limits the possibility of deploying such strategies on a large-scale under commercial conditions.

The current study will investigate the potential of in ovo-fed L-Leu, L-Met, and L-Cys combinations to alleviate the harmful effects of heat stress on broiler production leveraging in ovo technology currently available in commercial hatcheries. In this study, the effects of these amino acids on performance parameters, including growth, feed efficiency, and mortality rate under severe cyclical heat stress during the finisher phase were investigated. Furthermore, the hypothermic and antioxidant effects of these amino acids under the same stress conditions were also studied. We hypothesized that the hypothermic effects of L-Leu, in combination with the antioxidant capacities of L-Met and L-Cys, would reduce the detrimental effect of severe cyclical heat stress during the finisher phase.

## MATERIALS AND METHODS

This study was conducted at the Deschambault Research Center in Animal Science (Deschambault, Quebec, Canada) in accordance with the guidelines of the Canadian Council on Animal Care and was approved by the Institutional Animal Care and Use Committee of Université Laval (protocol number 2022-1016 approved on the 19th of April 2022).

### Experimental Treatments

This study was conducted between January and March of 2023. A total of 1,400 fertile eggs were obtained from a flock of Ross 308 broiler breeders (60 wk of age) and incubated under optimal conditions (37°C and 55% of relative humidity) from ED 1 to ED 18 in a multistage incubator without any intervention or manipulation (Scott Hatchery, Scott, Quebec, Canada). On ED 18, 5 solutions were prepared, and each one was injected into 280 eggs chosen at random. The solutions included a control group (**CTRL**, 52 µL of sterile diluent/egg), **T1** (1.0 mg of L-Leu/52 µL of sterile diluent/egg), **T2** (0.45 mg of L-Leu + 1.15 mg of L-Met/52 µL of sterile diluent/egg), **T3** (3.0 mg of L-Met + 2.0 mg of L-Cys/52 µL of sterile diluent/egg), and **T4** (0.40 mg of L-Leu + 1.60 mg of L-Met + 1.60 mg of L-Cys/52 µL of sterile diluent/egg). The sterile diluent used to prepare the treatments was the solution used at the hatchery to prepare vaccine solutions destined for in ovo vaccination (Boehringer Ingelheim Animal Health, Ontario, Canada). All amino acids were purchased from Sigma-Aldrich (L8912, M5308 and C7352 for L-Leu, L-Met and L-Cys, respectively). These doses were based on solution saturation with AA (i.e., the highest doses or combination of doses that can be delivered in the maximal volume of 52 µL that can be injected using the in ovo robot). Nevertheless, the amino acid ratios used were in accordance with literature (Han et al., 2017; Elwan et al., 2019). The in ovo treatments were injected into the eggs in the amniotic fluid through the air chamber using an in ovo injection robot (Embrex Inovoject JW84 System, Zoetis, Canada). On the day of hatch, hatched chicks were counted, wing-sexed, vaccinated against infectious bronchitis by spray (Massachusetts-type strain), and placed in pre-identified transportation boxes according to treatment group replicates. Hatch data is presented in Table 1.

**Table 1:** Hatch data per treatment group.^1^

Treatment^2^	Males	Females	Total hatch	Culled
CTRL	43.12	52.90	96.01	3.99
T1	45.49	54.17	99.65	0.35
T2	50.18	48.07	98.25	1.75
T3	48.21	47.50	95.71	4.29
T4	48.16	49.83	97.99	2.01

1 Data is expressed as percentages.

2 CTRL: 52 µL of sterile diluent/egg, T1: CTRL + 1.0 mg of L-Leu, T2: CTRL + 0.45 mg of L-Leu + 1.15 mg of L-Met, T3: CTRL + 3.0 mg of L-Met + 2.0 mg of L-Cys, T4: CTRL + 0.40 mg of L-Leu + 1.60 mg of L-Met + 1.60 mg of L-Cys.

### Experimental Design and Housing

Next, sexed chicks were transported over 90 kilometers to the Deschambault Research Center in Animal Science (Deschambault, Quebec, Canada) and treatment group replicates were placed in a broiler house according to a complete randomized block design consisting of 50 pens distributed in 2 rooms: a control or thermoneutral room (5 blocks of 5 replicates for a total of 25 pens of 28 birds/pen) and a heat stress room (5 blocks of 5 replicates for a total of 25 pens of 28 birds/pen). The pens were each equipped with a manual feeder and a bell drinker and were bedded with sawdust. Female birds were individually identified using a coloring agent that was applied to their back feathers. Males and females were distributed into the pens to achieve a 50 to 50% sex ratio in most pens to simulate mixed-sex rearing. In both rooms, ambient temperature was maintained at 33°C during the first week and was gradually reduced to 24°C by the end of the third week and was then maintained at 24°C until d 29. In the control (thermoneutral) room, the ambient temperature and relative humidity were maintained at 24°C and 45% respectively until d 34, while in the heat stress room, cyclical heat stress was applied by increasing the ambient temperature to 34°C and the relative humidity to between 55 and 60% from 8:00 am to 4:00 pm between d 29 and d 34. In the heat stress room, the relative humidity was maintained in the above-mentioned range using a fogging system. The choice of cyclical heat stress aimed to mimic the natural daily temperature variations between day and night. Humidity and temperature loggers (n = 3/room) were used to record these 2 parameters throughout the stress period (RuuviTag Bluetooth Sensor, Finland) and the average hourly temperature and relative humidity across the stress period were recorded.

### Measurements

**Figure 1:**
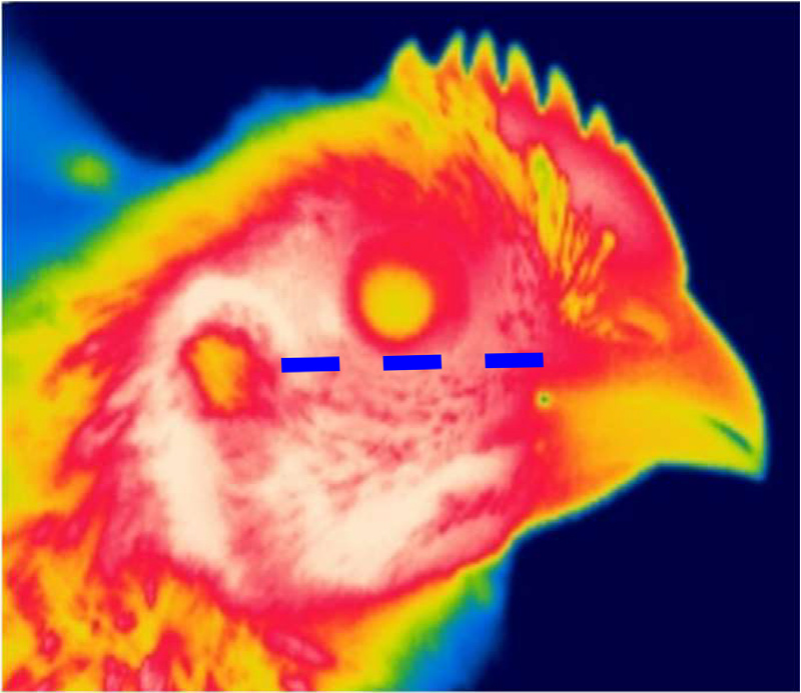
A thermal image illustrating the anatomical position on which the facial temperature was measured during the heat stress phase from d 29 to d 34. The dashed blue line highlights the zone from which the temperature was extracted.

During the experiment, birds had access to a standard broiler starter (Table 2), grower (Table 3), and finisher (Table 4) diets formulated to meet the Ross 308 recommendations, and to water ad libitum. Body weight and feed consumption were recorded on d 10, 21, and 35, while mortality was recorded on a daily basis. During the stress period (d 29–34), bird temperature was recorded twice per day (morning and afternoon) in the stress room and once per day (morning) in the control room using an infrared thermal camera (model T420bx, Teledyne FLIR Systems) on 2 males and 2 females per pen and per room (n = 4 birds × 2 measurements × 5 pens × 2 rooms = 80 data points/treatment/d). We choose to utilize a thermal camera to measure bird body temperature since it is less invasive, requires less bird manipulation, and temperatures obtained from thermal images have been shown to be very strongly correlated with core body temperature (Bloch et al., 2020). Thermal images were then processed using the FLIR Thermal Studio Suite Starter software (Teledyne FLIR Systems) to extract the facial temperature. The temperature was consistently determined from the same anatomical position on all images (Figure 1) and the average daily facial temperature was reported as the final body temperature.

**Table 2:** Ingredient and formulated nutrient composition of the starter diet (d 1–10).

Ingredient Name	Amount (kg)
Corn	424.11
Soybean meal	248.0
Wheat	150.0
Soya trituro, 44%	100.0
Meat and bone meal	50.0
Animal fat	7.0
DL-Methionine, 99%	4.34
Lysine sulfate, 70%	3.6
Lime stone	3.2
Salt	2.7
Threonine, 98.5%	1.86
Sodium bicarbonate	1.2
Vit. E, 50,000UI	1.0
Trace minerals premix	1.0
Liquid choline, 75%	0.61
L-Valine, 97%	0.52
L-Isoleucine, 98.5%	0.46
Vitamin Premix	0.30
Phytase	0.10
**Nutrient**	**Amount**
Crude protein, %	23.71
EMAn, kcal/kg	2972.80
DEB^1^, Meq/kg	254.0
dLys, %	1.31
dMet&dCys/dLys	0.76
dMet/dLys	0.56
dLeu/dLys	1.19

1 Dietary electrolyte balance.

**Table 3:** Ingredient and formulated nutrient composition of the grower diet (d 11–21).

Ingredient name	Amount (kg)
Corn	417.57
Soybean meal	238.0
Wheat	200.0
Soya trituro, 44%	75.0
Meat and bone meal	34.0
Animal fat	17.0
DL-Methionine, 99%	3.84
Lysine sulfate, 70%	3.0
Salt	2.8
Lime stone	2.6
Threonine, 98.5%	1.5
Sodium bicarbonate	1.4
Trace minerals premix	1.0
Vit. E 50,000 UI	0.7
Liquid choline, 75%	0.61
L-Valine, 97%	0.38
L-Isoleucine, 98.5%	0.30
Vitamin premix	0.20
Phytase	0.10
**Nutrient**	**Amount**
Crude protein, %	21.67
EMAn, kcal/kg	3049.4
DEB^1^, Meq/kg	238.0
dLys, %	1.17
dMet&dCys/dLys	0.78
dMet/dLys	0.56
dLeu/dLys	1.24

1 Dietary electrolyte balance.

**Table 4:** Ingredient and formulated nutrient composition of the finisher diet (d 22–34).

Ingredient Name	Amount (kg)
Corn	473.63
Wheat	200.0
Soybean meal	168.0
Soy trituro, 44%	100.0
Meat and bone meal	23.0
Animal fat	14.0
Lime stone	4.0
DL-Methionine, 99%	3.66
Lysine sulfate, 70%	3.60
Salt	2.80
Sodium bicarbonate	1.60
Threonine, 98.5%	1.52
Trace minerals premix	0.90
L-Isoleucine, 98.5%	0.70
Vit. E 50,000 UI	0.60
L-Arginine, 96.5%	0.59
L-Valine, 97%	0.58
Liquid choline, 75%	0.54
Vitamin premix	0.18
Phytase	0.10
**Nutrient**	**Amount**
Crude protein, %	19.5
EMAn, kcal/kg	3099.4
DEB^1^, Meq/kg	217.0
dLys, %	1.08
dMet&dCys/dLys	0.79
dMet/dLys	0.57
dLeu/dLys	1.24

1 Dietary electrolyte balance.

### Sample Collection and Lipid Peroxidation

On d 34, the heat stress phase was concluded, and all birds were returned to thermoneutral conditions (24°C and 45% relative humidity). On d 35 (during the phase of recovery from the effect of heat stress), blood samples (4 mL/bird) were taken from the brachial vein of 6 males and 6 females per in ovo treatment and per room (n = 120 birds in total). These samples were placed in tubes (BD Vacutainer 367,884 Lithium heparin, Franklin Lakes, NJ) on ice until they were returned to the laboratory (Department of Animal Science, Université Laval, Quebec, Canada) where they were centrifugated at 1,100 × g for 10 min at 4°C before extracting the plasma and storing it at -80°C until analysis. Sampled birds were then euthanized by cervical dislocation and samples (2–3 g) of the liver were taken, placed on dry ice, transported to the laboratory and stored at -80°C until analysis. The concentration of MDA in plasma and liver samples were then measured as described previously (Sammari et al., 2023) with modifications. Briefly, to increase MDA detection, liver samples were directly homogenized (Ika T18 Digital ULTRA-TURRAX, Germany) in a trichloroacetic acid buffer (30% w/v) with butylated hydroxytoluene (0.88% v/v) for 60 seconds at 12,000 RPM. As for plasma samples, a preliminary alkaline hydrolysis step was required prior to acidic protein precipitation to release bound MDA in sample in accordance with Pilz et al. (2000). This was achieved by combining 100 μL of plasma (or standard or H_2_O as a blank) with 25 μL of 3M NaOH, which was then capped, vortexed, and placed in a water bath (60°C for 30 min). Acidic protein precipitation was then performed by adding 1 mL of sulfuric acid (0.05 M) and 0.5 mL trichloroacetic acid (20% w/v).

### Statistical Analysis

The complete randomized block design was analyzed using a linear mixed effects model as implemented in the *lmerTest* package of R (Kuznetsova et al., 2017). For all collected data, the model included the block intra-room as a random effect. When an individual bird was the sampling unit (facial temperature, mortality rate), a pen-intra block was also added to the model as a random effect to account for variation in environmental conditions between blocks and pens. As for the fixed effects, the model included the effect of treatment, sex, and their interaction when the individual was the sampling unit, and only the treatment when the pen was the sampling unit. For data collected during the stress period, an additional thermal condition effect (control, stress) was also included in the model as a fixed effect. For facial temperature data recorded during the stress period, a day-of-stress effect was also included in the model as a fixed effect to account for daily variations in temperature. Results were presented as least squares means ± their standard errors. Pairwise comparisons between modalities of fixed effects were adjusted for multiple comparisons using Tukey adjustment as implemented in the *emmeans* package of R (R R Core Team, 2020). Preplanned contrasts corresponding to specific comparisons between treatment groups were also tested for significance using the *contrast* function of the *emmeans* package. For these contrasts, the CTRL group under thermoneutral conditions was considered the reference group. The contrasts consisted in comparing heat stressed groups (CTRL, T3 and T4) to the reference group. Differences between means were declared significant at *P* ≤ 0.05 and tendency was declared at 0.05 < P ≤ 0.10.

## RESULTS

### Effect of in Ovo-Fed Amino Acids on Performance

**Figure 2:**
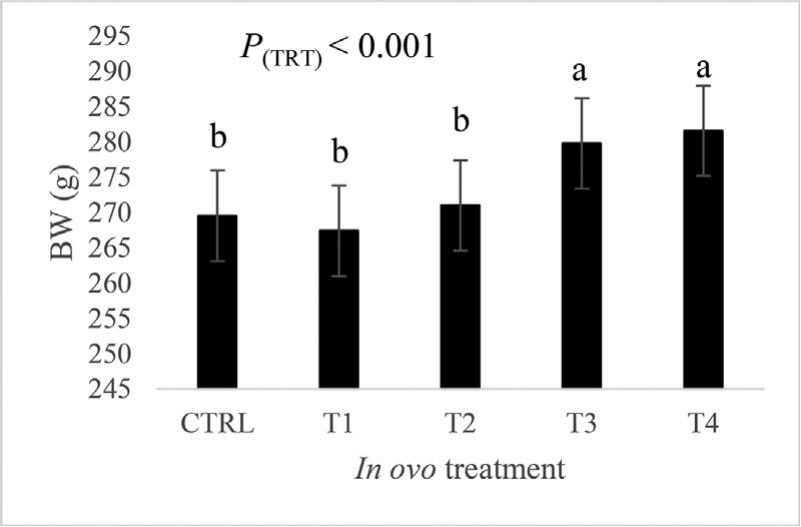
Effect of experimental in ovo treatments on body weight at d 10 (least squares means ± standard error). Means with uncommon letters (a–b) are significantly different at *P* < 0.05. CTRL: 52 µL of sterile diluent/egg, T1: CTRL + 1.0 mg of Leu, T2: CTRL + 0.45 mg of Leu + 1.15 mg of Met, T3: CTRL + 3.0 mg of Met + 2.0 mg of Cys, T4: CTRL + 0.40 mg of Leu + 1.60 mg of Met + 1.60 mg of Cys.

During the starter phase (d 1–10), birds BW was influenced by the in ovo treatment effect (*P* < 0.001). As depicted in Figure 2, BW of birds that received in ovo treatment T3 and T4 were significantly higher than all other treatment groups including the CTRL group (average of +10.0 g and +12.1 g for T3 and T4 relative to CTRL, respectively).

**Figure 3:**
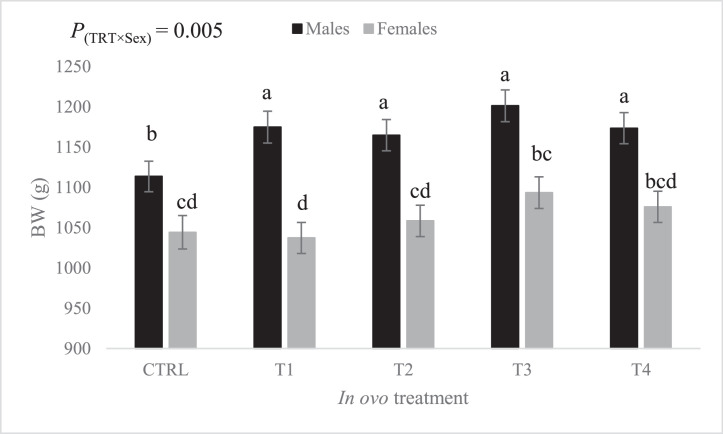
Effect of the interaction between *in ovo* treatment and sex on body weight at d 21 (least squares means ± standard error). Means with uncommon letters (a–d) are significantly different at *P* < 0.05. CTRL: 52 µL of sterile diluent/egg, T1: CTRL + 1.0 mg of Leu, T2: CTRL + 0.45 mg of Leu + 1.15 mg of Met, T3: CTRL + 3.0 mg of Met + 2.0 mg of Cys, T4: CTRL + 0.40 mg of Leu + 1.60 mg of Met + 1.60 mg of Cys.

During the grower phase (d 11–21), the effect of the treatment-by-sex interaction on BW (Figure 3) was statistically significant (*P* = 0.005). Males originating from eggs subjected to amino acids injection had a greater BW compared to females from all treatment groups. However, BW of females hatched from eggs injected with T3 and T4 did not differ significantly from BW of males CTRL treatment. In males, all in ovo treatments induced a significant increase in BW compared with the male CTRL treatment. In females, BW of birds that received the in ovo-injected amino acids were not significantly different from those of the female CTRL treatment.

**Figure 4:**
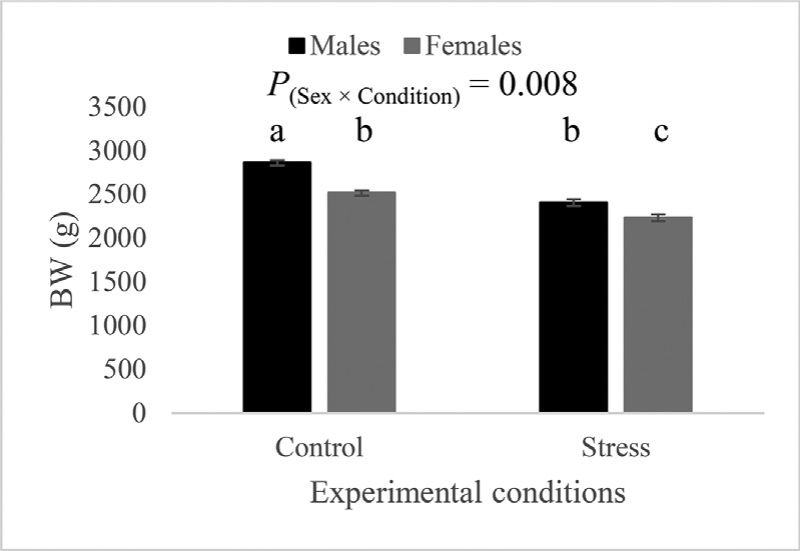
Effect of the sex-by-condition interaction on body weight at d 35 (least squares means ± standard error). Means with uncommon letters (a–c) are significantly different at *P* < 0.05. Control: 24°C and 45% relative humidity, Stress: 34°C and 55 to 60% relative humidity.

**Figure 5:**
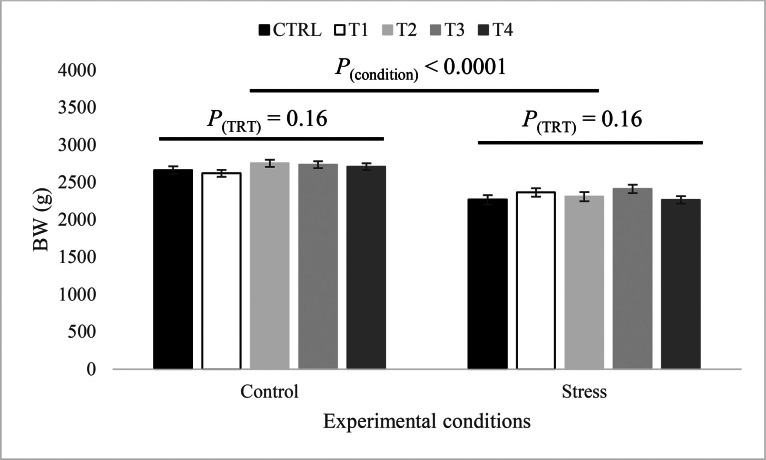
Effect of in ovo treatment and condition on body weight at d 35 (least squares means ± standard error). Means with uncommon letters (a–b) are significantly different at *P* < 0.05. Control: 24°C and 45% relative humidity, Stress: 34°C and 55 to 60% relative humidity. CTRL: 52 µL of sterile diluent/egg, T1: CTRL + 1.0 mg of Leu, T2: CTRL + 0.45 mg of Leu + 1.15 mg of Met, T3: CTRL + 3.0 mg of Met + 2.0 mg of Cys, T4: CTRL + 0.40 mg of Leu + 1.60 mg of Met + 1.60 mg of Cys.

**Figure 6:**
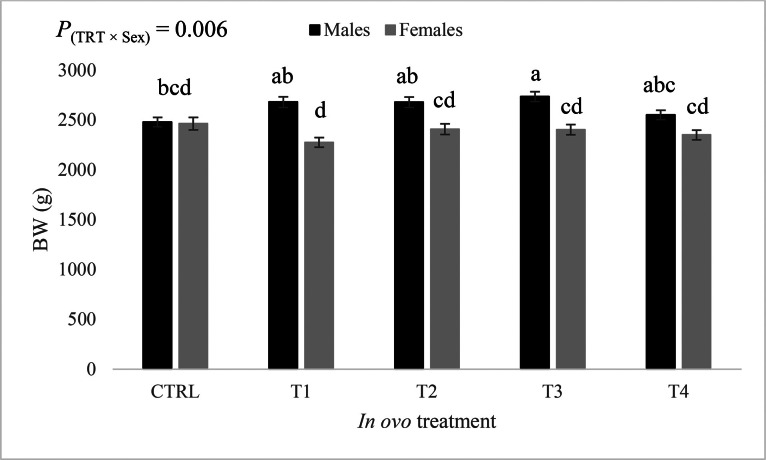
Effect of the sex-by- in ovo treatment interaction on body weight at d 35 (least squares means ± standard error). Means with uncommon letters (a–d) are significantly different at *P* < 0.05. CTRL: 52 µL of sterile diluent/egg, T1: CTRL + 1.0 mg of Leu, T2: CTRL + 0.45 mg of Leu + 1.15 mg of Met, T3: CTRL + 3.0 mg of Met + 2.0 mg of Cys, T4: CTRL + 0.40 mg of Leu + 1.60 mg of Met + 1.60 mg of Cys.

During the finisher phase (d 22–34), the effect of the sex-by-thermal condition interaction on BW was statistically significant (*P* = 0.008). As illustrated in Figure 4, the application of the heat stress led to a significant reduction in BW for both males and females. However, the impact was more pronounced in males than in females (-15.9 and -11.2% in males and females, respectively). The treatment effect on BW was not significant under both control and heat stress conditions (Figure 5). However, the sex-by-treatment interaction was significant (*P* = 0.006). Males and females from the CTRL treatment had similar BW, while males of group T1, T2, and T3 had significantly higher BW than their female counterparts (Figure 6). Additionally, males of treatment group T3 had significantly higher BW compared to the CTRL treatment, which was not the case for females.

With regard to feed efficiency (Table 5), birds from the experimental treatment groups had similar average daily feed intake (**ADFI**) compared to the CTRL group during the starter phase, except for the T1 treatment group, which had a lower ADFI (*P* = 0.03) compared with the CTRL treatment. Only birds from the T4 treatment group had a lower feed conversion ratio (**FCR**) than the CTRL group during this same phase (*P* = 0.03). In the grower phase, no significant differences were found between treatment groups in terms of ADFI or FCR. During the finisher phase, the interaction between in ovo treatment and thermal condition was not significant, neither for ADFI nor for FCR. The application of the cyclical heat stress significantly decreased ADFI and increased FCR in all treatment groups. As for the in ovo treatment effect, no significant differences were found between treatment groups in ADFI under thermoneutral conditions, while under heat stress conditions, treatment group T1 had a significantly lower ADFI than treatment group T3 but neither group was not significantly different from CTRL. In terms of FCR, the in ovo treatment effect was not statistically significant, and birds exposed to cyclical heat stress had significantly higher FCR (1.93 ± 0.04 vs. 1.78 ± 0.04).

**Table 5:** Effect of treatment, condition and their interaction on the average daily feed intake (ADFI) and feed conversion ratio (**FCR**) in the starter (d 1–10), the grower (d 11–21) and the finisher (d 22–35) phases.

Phase (d)	Parameter	Condition^2^	Treatment^1^					*P*-value		
			CTRL	T1	T2	T3	T4	TRT	Condition	I^3^
1 - 10	ADFI, g/d	-	28.8 ± 0.44a	26.8 ± 0.44b	27.1 ± 0.44ab	27.1 ± 0.47ab	27.1 ± 0.44ab	0.03	-	-
	FCR	-	1.039 ± 0.02a	1.024 ± 0.01ab	1.022 ± 0.01ab	0.996 ± 0.02ab	0.966 ± 0.01b	0.03	-	-
11 - 21	ADFI, g/d	-	97.9 ± 3.81	96.4 ± 3.81	97.0 ± 3.81	110.6 ± 3.81	101.5 ± 3.81	0.09	-	-
	FCR	-	1.33 ± 0.03	1.29 ± 0.02	1.27 ± 0.02	1.30 ± 0.02	1.33 ± 0.02	0.30	-	-
22 - 35	ADFI, g/d	Control	196.0 ± 4.47	184.0 ± 4.47	197.0 ± 4.47	203.0 ± 4.47	195.0 ± 4.47	0.002	< 0.0001	0.37
		Stress	160.0 ± 4.47ab	164.0 ± 4.47b	166.0 ± 4.47ab	183.0 ± 4.47a	172.0 ± 4.47ab			
	FCR	Control	1.85 ± 0.09	1.73 ± 0.09	1.77 ± 0.09	1.77 ± 0.09	1.82 ± 0.09	0.94	0.006	0.98
		Stress	1.99 ± 0.09	1.94 ± 0.09	1.92 ± 0.09	1.93 ± 0.09	1.89 ± 0.09			

1 CTRL: 52 µL of sterile diluent/egg, T1: CTRL + 1.0 mg of L-Leu, T2: CTRL + 0.45 mg of L-Leu + 1.15 mg of L-Met, T3: CTRL + 3.0 mg of L-Met + 2.0 mg of L-Cys, T4: CTRL + 0.40 mg of L-Leu + 1.60 mg of L-Met + 1.60 mg of L-Cys.

2 Control: 24°C and 45% relative humidity, Stress: heat stress 34°C and 55 to 60% relative humidity.

3 *P*-value of the interaction between treatment and condition.

As for mortality, the average weekly rate tended to be higher (*P* = 0.07) in males compared to females (1.06 ± 0.20 and 0.56 ± 0.20% for males and females, respectively) over the first 4 wk of the experiment before the application of the heat stress. During this same period, the effect of in ovo treatment on the mortality rate was not significant but the sex-by-treatment interaction (Table 6) tended to be significant (*P* = 0.054). In males, the numerically lowest weekly mortality rate was found in the CTRL and T4 treatment group, while in females, the numerically lowest weekly mortality rate was found in the T1 treatment group. In terms of cumulative mortality up to d 28 (Table 7), only the sex effect tended to be significant with males having higher overall mortality compared to females (4.0 ± 0.72 vs. 2.02 ± 0.72%). Numerically, treatment group T4 exhibited the lowest cumulative mortality rate of all treatment groups (1.99 ± 1.25, 3.98 ± 1.29, 4.07 ± 1.25, 4.13 ± 1.25 and 2.12 ± 1.25% for CTRL, T1, T2, T3, and T4, respectively).

**Table 6:** Effect of sex, treatment and their interaction on the average weekly mortality rate between d 1 and 28.

Sex	Treatment^1^					*P*-value		
	CTRL	T1	T2	T3	T4	Sex	TRT	I^2^
Males	0.26 ± 0.45	1.91 ± 0.47	1.57 ± 0.45	1.04 ± 0.45	0.52 ± 0.45	0.07	0.60	0.054
Females	0.73 ± 0.45	0.09 ± 0.45	1.57 ± 0.45	1.03 ± 0.45	0.54 ± 0.45			

1 CTRL: 52 µL of sterile diluent/egg, T1: CTRL + 1.0 mg of L-Leu, T2: CTRL + 0.45 mg of L-Leu + 1.15 mg of L-Met, T3: CTRL + 3.0 mg of L-Met + 2.0 mg of L-Cys, T4: CTRL + 0.40 mg of L-Leu + 1.60 mg of L-Met + 1.60 mg of L-Cys.

2 *P*-value of the interaction between sex and treatment.

**Table 7:** Effect of sex, treatment and their interaction on the cumulative mortality for the period between d 1 and 28.

Sex	Treatment^1^					*P*-value		
	CTRL	T1	T2	T3	T4	Sex	TRT	I^2^
Males	1.05 ± 1.77	7.59 ± 1.87	6.29 ± 1.77	4.14 ± 1.77	2.09 ± 1.77	0.057	0.52	0.13
Females	2.91 ± 1.77	0.37 ± 1.77	1.83 ± 1.77	4.11 ± 1.77	2.14 ± 1.77			

1 CTRL: 52 µL of sterile diluent/egg, T1: CTRL + 1.0 mg of L-Leu, T2: CTRL + 0.45 mg of L-Leu + 1.15 mg of L-Met, T3: CTRL + 3.0 mg of L-Met + 2.0 mg of L-Cys, T4: CTRL + 0.40 mg of L-Leu + 1.60 mg of L-Met + 1.60 mg of L-Cys.

2 *P*-value of the interaction between sex and treatment.

The exposure to thermal stress during the stress period (d 29–34) significantly increased the mortality rate (32.25 ± 5.1 vs. 0.92 ± 5.1%, *P* = 0.002). Moreover, male mortality rate was significantly higher than that of females (19.2 ± 3.7 vs. 14.0 ± 3.7%, *P* = 0.01). As for the interaction between sex and thermal condition, it only tended to be significant (*P* = 0.059) with a mortality rate not differing between males and females under control conditions (1.6 ± 5.24 and 0.22 ± 5.24, respectively), while under stress conditions, males had higher mortality rate than females (36.7 ± 5.24 and 27.7 ± 5.24, respectively). Finally, in regard to in ovo treatment effects, the effect of treatment-by-sex and treatment-by-condition interactions on mortality rate were not statistically significant. However, it is noteworthy that under stress conditions (Table 8), birds from the T4 treatment group exhibited numerically the lowest mortality rate of all treatment groups (51% and 54% of the mortality rate in the male and female CTRL groups, respectively).

**Table 8:** Effect of condition, sex, and treatment on the mortality (%) from d 29 to d 35.

Condition	Sex	Treatment^1^					*P*-value		
		CTRL	T1	T2	T3	T4	Condition	Sex	TRT
Control	Males	0.0 ± 9.74	3.54 ± 9.74	4.61 ± 9.74	0.0 ± 9.74	0.0 ± 9.74	0.002	0.01	0.73
	Females	0.0 ± 9.74	0.0 ± 9.74	0.0 ± 9.74	0.0 ± 9.74	1.33 ± 9.74			
Stress	Males	40.56 ± 9.74	36.51 ± 10.24	40.44 ± 9.74	28.57 ± 9.74	20.61 ± 9.74			
	Females	32.22 ± 9.74	21.41 ± 9.74	33.98 ± 9.74	27.91 ± 9.74	17.47 ± 9.74			

1 CTRL: 52 µL of sterile diluent/egg, T1: CTRL + 1.0 mg of L-Leu, T2: CTRL + 0.45 mg of L-Leu + 1.15 mg of L-Met, T3: CTRL + 3.0 mg of L-Met + 2.0 mg of L-Cys, T4: CTRL + 0.40 mg of L-Leu + 1.60 mg of L-Met + 1.60 mg of L-Cys.

### Effects of in Ovo-Fed Amino Acids on Body Temperature During the Stress Phase

**Figure 7:**
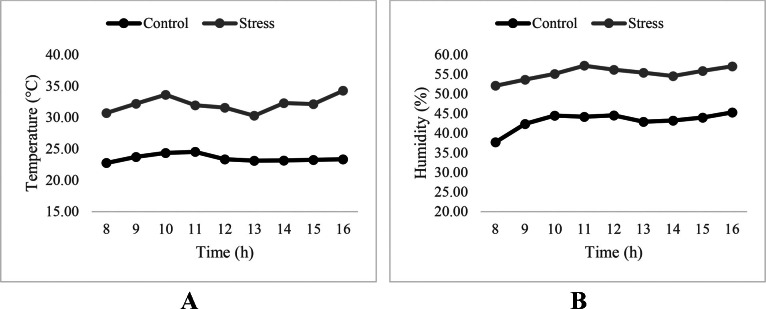
Average hourly ambient temperature (A) and relative humidity (B) in the control and stress rooms between d 29 and d 34. Control: 24°C and 45% relative humidity, Stress: 34°C and 55 to 60% relative humidity.

The average hourly ambient temperature and relative humidity in the control and heat stress rooms between d 29 and d 34 are illustrated on Figures 7A and 7B, respectively. The application of the cyclical thermal stress was successful as the target temperature (34°C) and relative humidity (55–60%) were achieved.

**Figure 8:**
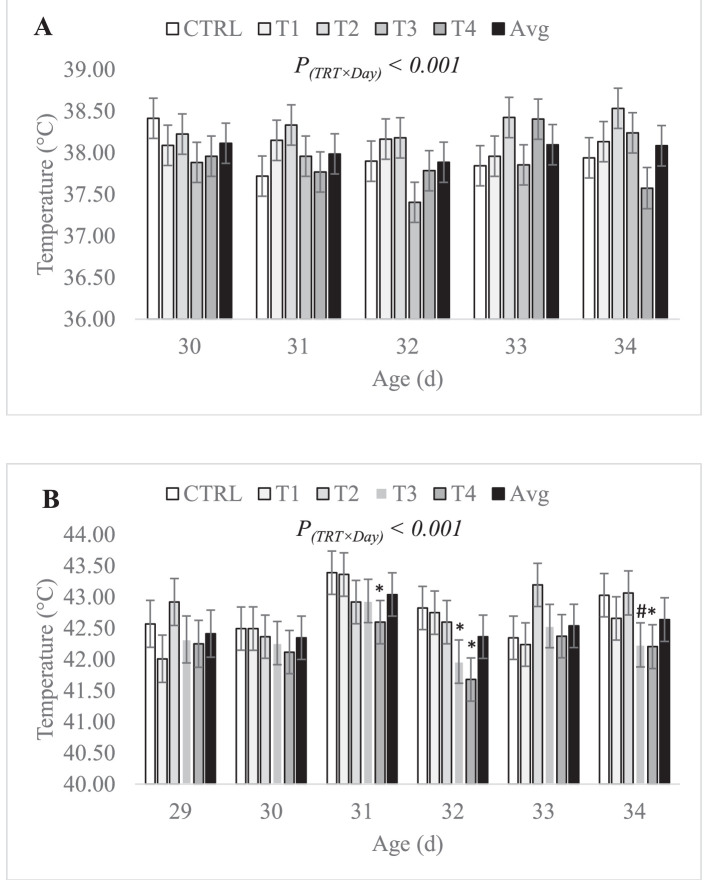
Effect of the in ovo treatment-by-day of stress interaction on birds’ facial temperature under control conditions (A) and stress conditions (B). Data is presented as least squares means ± standard error. CTRL: 52 µL of sterile diluent/egg, T1: CTRL + 1.0 mg of Leu, T2: CTRL + 0.45 mg of Leu + 1.15 mg of Met, T3: CTRL + 3.0 mg of Met + 2.0 mg of Cys, T4: CTRL + 0.40 mg of Leu + 1.60 mg of Met + 1.60 mg of Cys, Avg: average facial temperature. Control conditions: 24°C and 45% relative humidity, Stress conditions: 34°C and 55 to 60% relative humidity. *P(T3 or T4 vs. CTRL) ≤ 0.05, ^#^*P*(T3 or T4 vs. CTRL) < 0.1.

As expected, the statistical analysis revealed a significant effect of the thermal conditions on bird facial temperature (*P* < 0.0001), with birds kept under heat stress conditions having higher facial temperatures than those of birds kept under control conditions (42.6 ± 0.18 vs. 38.1 ± 0.18°C). Under both thermal conditions, the effect of the treatment-by-day interaction on facial temperature was significant (*P* < 0.001 and *P* < 0.001 under control and stress conditions, respectively). Under control conditions (Figure 8A), the intra-day differences in facial temperature between treatment means were not significant, but mean facial temperature of treatment groups varied significantly between d 30 and 34 (i.e., significant inter-day differences), which led to the significant interaction. On the other hand, the intra-day differences in facial temperature between treatment means, which are of interest, varied significantly under stress conditions (Figure 8B). In fact, the facial temperature of birds from treatment group T3 and T4 remained numerically under the average daily temperature of all treatment groups as well as the CTRL group throughout the entirety of the stress phase between d 29 and 34. Treatment group T4 exhibited a lower facial temperature as compared to the CTRL group on d 3 (-0.8°C, *P* = 0.05), on d 4 (-1.15°C, *P* = 0.007), and on d 6 (-0.82°C, *P* = 0.05) of the stress phase. As for treatment group T3, it exhibited the largest difference from the CTRL group on day 4 (-0.86°C, *P* = 0.04) and on day 6 (-0.79°C, *P* = 0.06) of the stress phase. Finally, the effect of sex on facial temperature was statistically significant under control conditions with males exhibiting higher facial temperatures than females (38.18 ± 0.09 vs. 37.98 ± 0.09°C, *P* < 0.001), while under stress conditions, the difference between sexes was no longer significant (42.63 ± 0.22 and 42.57 ± 0.22°C in males and females, respectively, *P* = 0.37).

### Effects of in Ovo-Fed Amino Acids on Lipid Peroxidation

**Figure 9:**
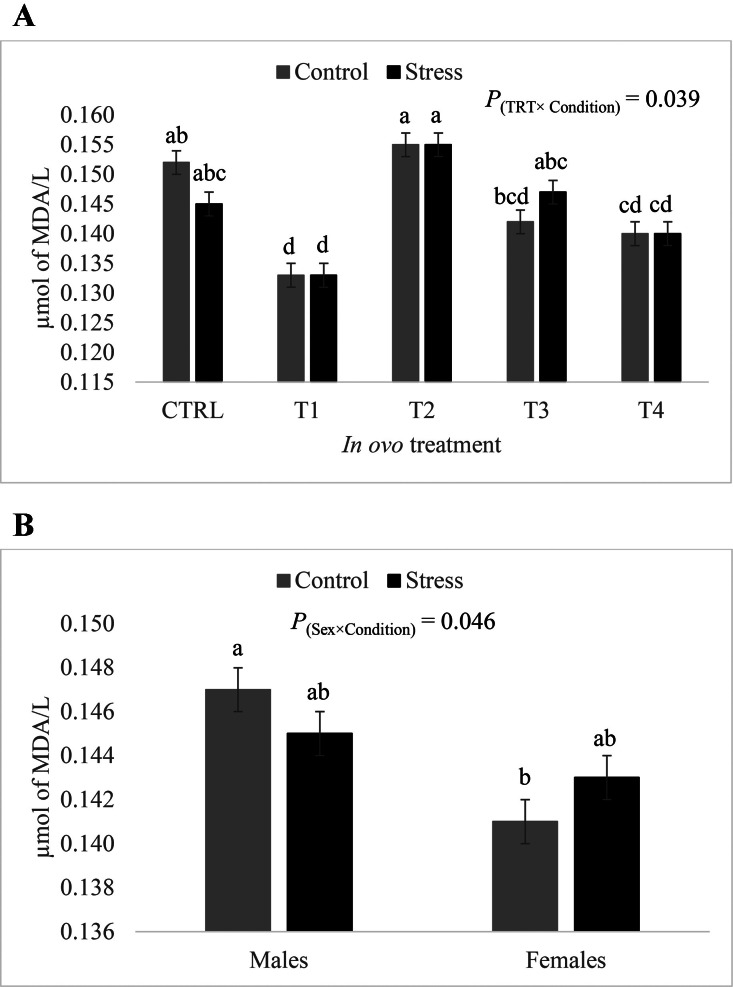
(A) Effect of the in ovo treatment-by-condition interaction on plasma malonaldehyde concentrations. Data is presented as least squares means ± standard error. Control: 24°C and 45% relative humidity, Stress: 34°C and 55 to 60% relative humidity. CTRL: 52 µL of sterile diluent/egg, T1: CTRL + 1.0 mg of Leu, T2: CTRL + 0.45 mg of Leu + 1.15 mg of Met, T3: CTRL + 3.0 mg of Met + 2.0 mg of Cys, T4: CTRL + 0.40 mg of Leu + 1.60 mg of Met + 1.60 mg of Cys. Means with uncommon letters (a–d) are significantly different at *P* < 0.05. (B) Effect of the sex-by-condition interaction on plasma malonaldehyde concentrations. Data is presented as least squares means ± standard error. Control: 24°C and 45% relative humidity, Stress: 34°C and 55 to 60% relative humidity. Means with uncommon letters (a–b) are significantly different at *P* < 0.05.

Our statistical analysis revealed significant treatment-by-thermal condition (*P* = 0.039) and sex-by-condition (*P* = 0.046) interactions for plasma MDA levels. As can be seen in Figure 9A, intra-group plasma MDA levels were very similar between control and stress conditions. The T1 treatment group exhibited significantly lower plasma MDA concentrations under both conditions as compared to the CTRL group. Plasma samples from the T4 treatment group, under both conditions, exhibited lower MDA concentrations than the CTRL group under control conditions (i.e., the reference group), but not under stress conditions, while treatment groups T2 and T3 did not differ significantly from the CTRL group under both conditions. Figure 9B illustrates the effect of the sex-by-condition interaction on plasma MDA concentrations. Under control conditions, MDA concentrations in the plasma were slightly but significantly higher in males than in females, while under stress conditions, males and females had similar plasma MDA concentrations.

**Figure 10:**
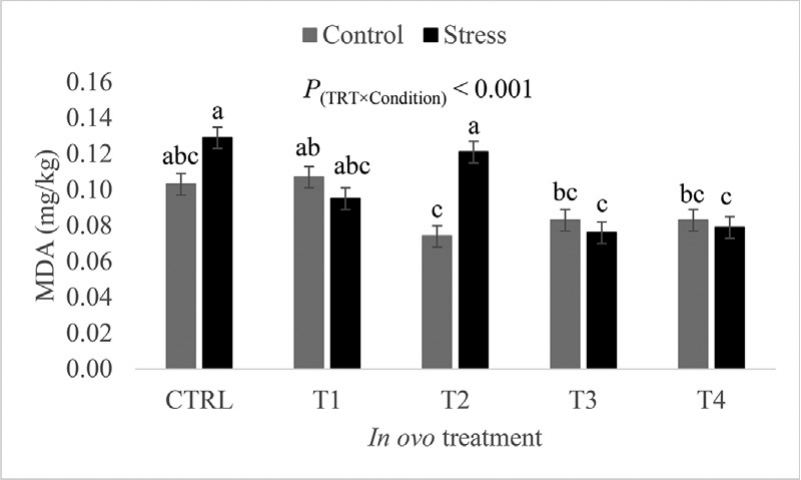
Effect of the in ovo treatment-by-condition interaction on malonaldehyde concentrations in the liver. Data is presented as least squares means ± standard error. Control: 24°C and 45% relative humidity, Stress: 34°C and 55 to 60% relative humidity. CTRL: 52 µL of sterile diluent/egg, T1: CTRL + 1.0 mg of Leu, T2: CTRL + 0.45 mg of Leu + 1.15 mg of Met, T3: CTRL + 3.0 mg of Met + 2.0 mg of Cys, T4: CTRL + 0.40 mg of Leu + 1.60 mg of Met + 1.60 mg of Cys. Means with uncommon letters (a–c) are significantly different at *P* < 0.05.

With regard to MDA concentrations in the liver, the thermal condition-by-in ovo treatment interaction was highly significant (*P* < 0.001). Under control conditions, MDA concentrations in the liver of birds from the treatment groups did not differ from that of birds from the CTRL group. In contrast, group T3 and T4 had MDA concentrations significantly lower than that of the CTRL group under heat stress conditions (Figure 10). The effect of sex (*P* = 0.24) and the effect of its interactions with the condition and with the in ovo treatment on liver MDA levels were not statistically significant in liver samples (data not shown).

## DISCUSSION

Heat stress represents a major challenge to the poultry industry worldwide. In light of its detrimental effects on broiler production, strategies that could improve thermotolerance in broiler chickens or alleviate the negative effects of high ambient temperatures are of great interest for the poultry industry. In ovo-fed amino acids including L-Leu, L-Met, and L-Cys have been shown to improve tolerance for high ambient temperatures and to improve the response of the antioxidant defense system during embryogenesis and early post-hatch (Han et al., 2017; Elnesr et al., 2019; Elwan et al., 2019; Han et al., 2019). However, very little is known about the effect of in ovo-fed amino acids on growth, FCR, body temperature, and mortality in the finisher phase of broiler chickens. Thus, the current study was designed to investigate the potential of BCAA and SAA fed by in ovo injection to alleviate the effect of heat stress on performance, body temperature, and oxidative stress during the finisher phase and under severe cyclical heat stress. To induce this heat stress, a combination of 34°C and 60% relative humidity was applied in the heat stress section of the poultry house (Figure 7). To maintain the relative humidity at this level in the HS section of the poultry house, a fogging system was used as indicated in the Materials and Methods section. This is important to highlight since the effect of HS discussed in the following sections was not solely due to increased ambient temperature. Rather, it was the result of the combined effects of elevated ambient temperature and relative humidity.

During the starter phase, the sex-by-in ovo treatment interaction for BW was not significant. Our analysis revealed a significant increase in BW at d 10 for birds that were in ovo-fed with L-Met and L-Cys (T3), L-Met + L-Cys + L-Leu (T4) compared to the injected control group (CTRL). One possible explanation for this increase in BW is that in ovo-fed L-Met and L-Cys have been shown to improve the antioxidant status of the small intestine of newly hatched broiler chicks exposed to heat stress during incubation (Elnesr et al., 2019). These amino acids have been shown to improve intestinal histomorphometric characteristics, such as increased intestinal surface area (Elwan et al., 2019). Such improvement could enhance digestion, nutrient absorption, and ultimately feed efficiency. This argument is supported by the significant reduction of FCR in the starter phase in birds that were in ovo-fed with the combination of L-Leu + L-Met + L-Cys (T4). In addition to the above-discussed effects on the intestinal tube, these amino acids could have also induced epigenetic modifications leading to the reported growth and FCR improvements. It has been shown that supplementing female broiler breeders’ diets with methionine was associated with a significant increase in BW on d 49 and in eviscerated carcass weight of their progeny (Liu et al., 2020b) suggesting and epigenetic effect of these amino acids.

During the grower phase, the effect of the in ovo treatment on BW differed between males and females. Males seemed to benefit equally from all in ovo treatments, as demonstrated by the significant increase in their BW compared to the injected control group. Male BW did not differ between treatment groups, but males of the T3 treatment group had numerically the highest BW (+7.9% relative to male CTRL group). In contrast, females seemed to no longer benefit from the in ovo treatments in terms of growth, as their BW remained similar to that of the injected control group. Moreover, females from the T3 group had numerically the highest BW (+4.7% relative to female CTRL group). This between-sex divergence in response to in ovo-fed AA has been previously reported (Han et al., 2018). Specifically, males were shown to benefit from in ovo-fed AA more than females in terms of their ability to adjust feed intake to reduce metabolic heat production and to acquire thermotolerance. However, the mechanisms underlying this sex-based response are still largely unknown and warrants further investigations. The less consistent and sex-dependent effect of in ovo treatment groups on BW after the starter phase could potentially suggest that they were metabolized and utilized differently in males than in females. However, this hypothesis needs to be experimentally validated in a future study. As for the numerically higher BW of birds from in ovo treatment T3, we observed a slight but not significant increase in their feed intake compared to all other treatment groups, which could explain the corresponding slight but not significant increase in their BW. A previous study has demonstrated that in ovo feeding of L-Cys was associated with increased feed intake and higher BW gain compared to the control group injected with distilled water (Ajayi et al., 2022).

Regarding the weekly mortality rate recorded before the heat stress phase, it remained below 2.0%, which is in line with mortality rates reported in the literature for fast-growing broiler strains under optimal conditions (Torrey et al., 2021). In terms of cumulative mortality between d 0 and d 28, while the between-treatment differences were not statistically significant, combining L-Leu, L-Met and L-Cys (T4) resulted in the numerically lowest cumulative rate of all treatment groups. This rate remained very close to 2.0%, similar to that of the CTRL group. The reasons behind the lower mortality rate in the T4 treatment group have not been fully clarified yet. However, it could be speculated that by stimulating growth (Ajayi et al., 2022), improving digestive tract structural integrity (Elwan et al., 2019; Ajayi et al., 2022), and enhancing immune response (Awad et al., 2020), this combination of injected aminos could decrease the mortality rate of birds hatched from treated eggs.

The application of the cyclical thermal stress during the finisher phase resulted in a significant decrease in feed intake and in BW, coupled with a significant increase in FCR. These changes in performance are mainly the result of increased body temperature under heat stress conditions. Reduced feed intake is a well-known consequence of heat stress (Awad et al., 2020; Emami et al., 2021). In fact, a recent meta-analysis estimated a 1.4% decrease in ADFI for each degree Celsius above the upper critical limit of the thermoneutral zone in broilers older than 21 d (Andretta et al., 2021). These authors also highlighted that 74% of decreases in BW gain under heat stress were directly related to this reduction in feed intake. This decrease in feed intake is an adaptive mechanism allowing birds to reduce their metabolic heat production under high ambient temperatures (Andretta et al., 2021). Heat stress has also been shown to alter the morphometric characteristics of the gastrointestinal tract leading to an increase in intestinal permeability (Tabler et al., 2020). More specifically, changes in permeability directly compromise intestinal integrity through tight junction disruption. This facilitates paracellular translocation of pathogenic bacteria and noxious toxins from the intestines into the blood stream, resulting in a condition known appropriately as leaky gut syndrome (Galarza-Seeber et al., 2016). This condition causes chronic systemic inflammation due to increased elicited host defense responses (Kogut et al., 2018). The chronic persistence of such inflammation is known to hinder poultry zootechnical performance (Ahmad et al., 2022).

Furthermore, heat stress also alters the secretion of several hormones from the enteroendocrine cells of the gastrointestinal tract involved in feed intake regulation, suggesting that high ambient temperatures may interfere with voluntary feed intake by altering bird appetite through varying levels of GI peptides and biogenic amines (Mazzoni et al., 2022). In the present study, a specific contrast comparing ADFI between the CTRL group under control conditions (i.e., reference group) and the CTRL group under heat stress conditions revealed an 18.4% (*P* < 0.001) decreased in ADFI. This decrease in feed intake reflects the multifaceted impact of heat stress. This effect could be mediated by various factors and their combination, including: 1) increased body temperature, which consequently reduces bird capacity to dissipate metabolic heat and thus commensurately decreases voluntary feed intake, 2) heat stress-induced oxidative damage, and 3) localized (e.g., small intestine) and systemic inflammatory processes triggered by heat stress.

Compared to the unstressed CTRL group (i.e., reference group), birds that were in ovo-fed a combination of L-Met and L-Cys (T3) and a combination of L-Met, L-Cys, and L-Leu (T4) seemed to be the least affected by heat stress in terms of ADFI. In greater details, the specific contrast comparing the heat stressed T3 and T4 treatment groups to the unstressed CTRL group showed a reduction of only 6.6% (*P* < 0.001) and 12.2% in ADFI (*P* < 0.01), respectively. These specific contrasts indicate that, in comparison to the stressed CTRL group, in ovo treatments T3 and T4 alleviated the effect of heat stress on ADFI by 12.56% (23.1 g/d) and 6.97% (12.2 g/d), respectively. However, the positive effect of these 2 in ovo treatment groups on ADFI was not enough to significantly reduce the effect of heat stress on BW.

With regard to mortality, a specific contrast comparing the stressed CTRL to the unstressed CTRL group revealed a 36.0% increase (*P* < 0.01) in mortality rate due to heat stress which is similar to mortality rate reported in a previous study in which a cyclical heat stress regimen (34°C for 6 h/d) was applied from d 22 to 35 (Awad et al., 2020). In addition to the physiological and metabolic consequences of hyperthermia and heat shock (Brugaletta et al., 2022), heat stress exerts a detrimental effect on the immune response. Specifically, it could reduce the proliferation and differentiation of lymphocytes (Hirakawa et al., 2020). Concurrently, heat stress diminishes the levels of circulating immune globulins (Awad et al., 2020). Furthermore, it contributes to an increase in harmful pathogens including coliforms and clostridia in the small intestine (Song et al., 2014), and to compromising the integrity of the small intestine by increasing its permeability (Nanto-Hara et al., 2020). This compromised integrity opens the way for systemic infections, which could contribute to an elevated mortality rate. Moreover, panting, a primary behavioral response to heat stress in poultry to increase evaporative heat loss, can induce dangerous acid-base dysregulation through respiratory alkalosis and metabolic acidosis, ultimately contributing to increased mortality (Wasti et al., 2020). Therefore, the combination of behavioral alteration, suppressed immune function, neuroendocrine dysregulation, and oxidative stress likely contributed to the reported mortality rate.

Similar to their effect on ADFI, in ovo treatments T3 and T4 alleviated the harmful effect of heat stress on mortality in comparison to the stressed CTRL group that was only injected with sterile diluent. More specifically, a contrast comparing the heat stressed T3 and T4 treatment groups to the reference group showed that mortality rate decreased to 28.2% (*P* = 0.03) and to 19% (*P* = 0.13), respectively. The lower mortality rate in the T3 and T4 treatment group compared to the reference group suggest that combinations of L-Met, L-Cys and L-Leu were beneficial in reducing the mortality in heat stressed broiler chickens. In fact, the difference in mortality between the stressed T4 and the reference group was not statistically significant as evidenced by the specific contrast comparing these 2 groups (*P* = 0.13). This finding confirms the favorable effect of the T4 in ovo treatment on mortality under heat stress. Although the underlying mechanisms of action are still to be elucidated, these 2 treatment groups (T3 and T4) were associated with below-average facial temperature during the stress phase (Figure 8B). Additionally, these 2 groups were the least affected by heat stress in terms of ADFI. This could have led to a better immune status in birds from these 2 groups given that diet-derived nutrients have a considerable impact on immunity (Kidd, 2004). Thus, it is plausible to suggest that the beneficial effects of these in ovo treatments for the mortality rate could be, at least partly, attributed to their hypothermic effect leading to less affected ADFI and subsequently a lower mortality compared to the CTRL group. This statement will be validated in a future study. Finally, the possibility of some birds adapting to the effect of heat stress as a result of repeated exposure to stress-recovery cycles, and hence surviving until the end of the experiment due to this ability to adapt, cannot be fully excluded. However, given the homogeneous genetic background of commercial broilers, the between-bird variability in terms of their ability to cope with the effect of heat stress would be expected to remain low.

In addition to reducing body temperature under heat stress, the antioxidant properties of L-Met, L-Cys, and L-Leu as well as their implication in the response of the antioxidant defense system are well documented (Han et al., 2022; Sarsour and Persia, 2022). In their study, Han et al. (2022) exposed Ross 308 broilers, hatched from eggs that were injected with 9.05 mg of L-Leu or with sterile water, to natural summer heat waves from d 21 to d 39. These authors showed that broilers from the L-Leu group had significantly higher serum glutathione peroxidase and lower serum MDA concentrations at d 39. In the present study, the T1 (L-Leu) group exhibited a significantly lower concentration of plasma MDA in comparison to the CTRL group under both control and stress conditions. This result is in accordance with findings from Han et al. (2022) despite variation in the injection site (yolk vs. amniotic fluid) and injection time (ED 7 vs. 18) between the 2 studies. Furthermore, treatment group T4 (L-Met + L-Cys + L-Leu) exhibited significantly lower plasma MDA concentration compared to the reference group, which is also in agreement with the above-cited literature, demonstrating the antioxidant properties of in ovo-fed SAA. Moreover, in the current study, MDA concentrations in the liver of heat stressed broilers from the T3 and T4 treatment groups were significantly lower than their respective CTRL group. This finding further supports the benefits of in ovo-fed BCAA and SAA in terms of alleviating oxidative damage in heat-stressed broilers. It is worth noting that intra-group MDA levels in the plasma were very similar under control and heat stress conditions, while in the liver, these levels varied depending on the condition. In this study, blood and liver samples were taken during the recovery phase of the stress cycle (i.e., during the time of day where heat stress was not applied). Thus, the similar intra-group plasma MDA levels under both conditions suggest that heat stress had a more lasting oxidative effect in the liver than in the blood. Hence, it is plausible that the plasma MDA concentration in heat-stressed birds decreased to similar levels of birds kept under thermoneutral conditions during the recovery phase.

The combined hypothermic and antioxidant effects of BCAA and SAA likely contributed to the diminished impact of heat stress on ADFI and mortality rates in the T3 and T4 treatment groups, as previously discussed. In the present study, although the statistical models fitted to the data accounted for the room effect as both a random and a fixed effect, it cannot be fully excluded that some minor portion of the effects observed in this study could be potentially attributed to variation between rooms that the statistical models could not capture due to the complexity of the experimental design.

## CONCLUSIONS

Heat stress continues to pose a major challenge for the poultry industry, especially in light of the predicted increases in surface temperatures if no effective climate action is taken in the near future. Current strategies used by poultry producers to alleviate the harmful effects of heat stress on their birds are based on the use of dynamic ventilation systems coupled with cooling systems, as well as on various nutritional strategies. An additional strategy, based on improving bird thermotolerance, can complement these existing strategies and further reduce the detrimental effects of heat stress on poultry production.

The present study is the first in a series of studies that aim to explore the potential of in ovo-fed amino acids according to commercial hatchery practices to enhance thermotolerance in broiler chickens. Here, we showed that in ovo-fed BCAA (L-Leu) and SAA (L-Met and L-Cys) had a positive impact on BW and FCR during the starter phase. We also showed that the positive effect of these amino acids on BW continued during the grower phase especially in male birds. As for the finisher phase, we demonstrated that these amino acids had the potential to reduce the impact of severe cyclical heat stress on some important performance parameters (e.g., ADFI and mortality rate) through their combined hypothermic and antioxidant effects leading to a significant decrease in body temperature and in oxidative damage under heat stress conditions. In a future study, the mechanism of action of these in ovo-fed amino acids will be investigated using a multi-omics approach combining metabolomics and transcriptomics of vital internal organs affected by heat stress.

## DISCLOSURES

The authors declare no conflicts of interest.
